# Mobile Applications in Evaluations of Knee Joint Kinematics: A Pilot Study

**DOI:** 10.3390/s19173675

**Published:** 2019-08-23

**Authors:** Przemysław Lisiński, Agnieszka Wareńczak, Krystyna Hejdysz, Paweł Sip, Jarosław Gośliński, Piotr Owczarek, Justyna Jonak, Jagoda Goślińska

**Affiliations:** 1 Department of Rehabilitation and Physiotherapy, Poznan University of Medical Sciences, 28 Czerwca 1956 Str., No 135/147, 60-545 Poznań, Poland; 2 Aisens Sp. z o. o., Lubeckiego 23A, 60-348 Poznań, Poland; 3 Department of Politics and International Relations, University of Southampton, Southampton SO17 1BJ, UK

**Keywords:** wireless sensors, mobile applications, movement, knee joint, telerehabilitation

## Abstract

Because medical professionals lack the means to monitor exercises performed by patients in their home environment directly, there is a strong case for introducing technological solutions into this domain. They include methods that use wireless inertial sensors, which emit signals recorded and processed by special applications that work with mobile devices. This paper’s aim is (a) to evaluate whether such sensors are suitable for qualitative and quantitative motion analysis, and (b) to determine the repeatability of results over a few recordings. Knee joint activity was analysed using a system of inertial sensors connected through a Wi-Fi network to mobile devices. The tested individuals did eight different activities, all of which engaged the knee joint. Each excercise was repeated three times. Study results did not reveal any statistically significant differences between the three measurements for most of the studied parameters. Furthermore, in almost every case, there were no statistically significant differences between the results of the right and left lower limb (*p* > 0.05). This study shows that easy use and repeatability of results combined with the feature of quantitative and qualitative analysis make the examined method useful for functional evaluations of the knee joint.

## 1. Introduction

For patients with reduced mobility, treatment effects largely depend on active engagement in the therapeutic process [1]. This is because under many rehabilitation procedures, patients are required to conduct active exercises without supervision, whose role is to, among other things, decrease pain levels, improve a given joint’s motion range [2], increase muscle strength [3], and finally, enhance motor coordination and movement precision [4]. For both in-patient and out-patient rehabilitation centres, the methodological correctness of exercise performance (technique, intensity, frequency) is monitored directly during therapeutic sessions by physical medicine and rehabilitation specialists. This is not possible, however, when after methodological instruction, the patient is tasked with exercising independently in their dwelling. Even when exercise performance is overseen at a treatment centre, there is still often the need for patients to do exercises on their own, outside the therapeutic setting. In clinical practice, treatment evaluation essentially means applying simple methods to measure, for instance, pain intensity using the visual analogue scale (VAS) [5], ranges of active and passive motion with mechanical or electrical goniometers [6], muscle strength with the manual muscle testing (MMT) scale [7], or motor coordination and precision with functional test methods [8,9]. Although all these methods are prevalent in clinical practice due to their simplicity, they are also often characterised by an inevitable imperfection as there is an element of subjectivity to them [7,10]. Therefore, we concluded that there is a strong need for developing a method for monitoring exercise correctness outside of clinics. Such a technique should exhibit acceptable measurement accuracy, repeatability of results, versatile and simple applications, and, most crucially, it should enable a therapist to continually monitor the correctness of independently-performed exercises; a need that has been indicated in merely a few pieces of relevant literature [11,12]. At the same time, it is worth noting that the dynamic growth of information technology (IT) techniques in hardware and software, together with developments in availability and speed of online information transfer, theoretically enable a physician or a therapist to oversee patients’ exercise quality. At this point, a question arises as to which methodological aspects of technical and IT solutions have already been implemented into rehabilitation treatment? After carefully analysing the available literature, we were able to find mainly articles describing the use of vision and inertial systems in telerehabilitation, which until now were primarily in the form of games, making rehabilitation more attractive to patients [13,14]. Currently, telerehabilitation tools are being created which not only encourage patients to exercise, but also enable specialists to collect results related to parameters such as ranges of motion or speed of performed exercises. Then, it will be possible to monitor the improvement process and catch the errors during home exercises [13,15]. One of the available tools on the market are wireless inertial sensors attached to a patient’s body, which emit signals that are recorded, analysed, and processed by special mobile applications [16,17]. It seems that the ease of use of such sensors may affect that they will become a leading solution in telerehabilitation.

Aim of the Study: At this stage of system assessment, this paper aims to determine the validity of using Orthyo sensors in connection with the Orthyo-App mobile application as a tool for monitoring selected, most commonly prescribed knee joint activities. This study investigated the system’s ability to simultaneously track selected kinematic parameters, as well as the repeatability of obtained values. To this end, three measurements were taken for each of the examined activity and in the same testing conditions. Such set-up was employed to demonstrate the presence or lack of any possible significant differences between results.

## 2. Materials and Methods

### 2.1. Participants

Initally, 45 female students of physiotherapy at Poznan University of Medical Sciences volunteered to participate in the study. They were informed about the aims and methodology, and they provided written consent to participate in the project. The mean age was 22.8 ± 2.4, height 167.2 ± 5.6 cm, body mass 62.4 ± 8.2 kg, and body mass index (BMI) 22.3 ± 2.6. All of the individuals in the group showed good health status. The participants shared similar physical activity habits, both in frequency and type of performed sports. The following exclusion criteria were adopted: Recent injuries and surgical treatment of lower extremities that could affect knee joint function; the presence of pain anywhere in the body, limiting day-to-day activity; and neurological, cardiovascular, and rheumatic diseases impacting patients’ function. Based on adopted inclusion and exclusion criteria and the need to form a homogeneous group in terms of health status and physical activity, the study finally encompassed 30 individuals.

### 2.2. Sensors and Application

**Figure 1:**
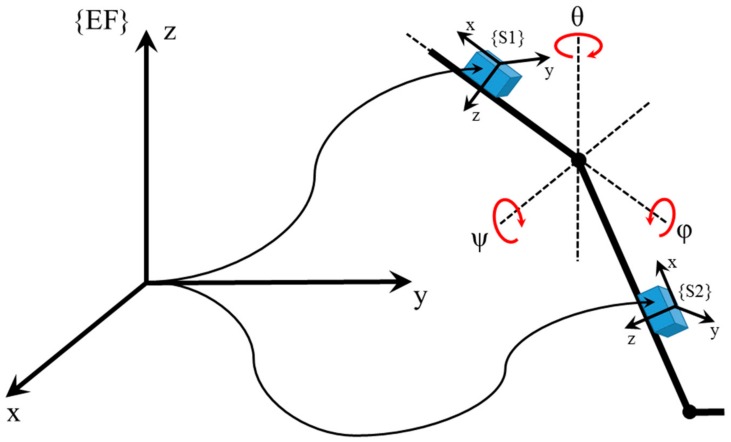
The coordinate system of the sensory system used in the experiment.

The Orthyo system (Aisens sp.z o.o. Poznan, Poland) is a certified medical device class Im (I measure) according to classification of medical devices in Poland. The measurement system is approved by the Central Office of Measures in Poland. It uses three basic types of sensory data: inertial (linear acceleration and angular velocity), magnetic (magnetic induction vector), and environmental (atmospheric pressure and temperature). Output data are processed in sensors, and each of them is composed of an accelerometer, a gyroscope, a magnetometer, a barometer and a thermometer. Sensory data is first processed by a sensor’s microchip. At this stage, raw sensory data are filtred, calibrated, and then computed in an estimation process. Under the process, the sensors’ location in a referential system is established (a coordinate system whose axes are positioned following the east–north–up (ENU) principle, where X points eastwards, Y northwards, and Z upwards). In addition, the estimation algorithm can estimate the relative position (concerning the zero position, i.e., the moment after a sensor has been turned on). Estimation and calibration are run using such estimators as the Kalman filter, complementary filters, and supporting artificial intelligence (AI) algorithms. Such computed data is sent further via Wi-Fi/Bluetooth low-energy to the Orthyo-App. In the application, the second stage of data processing is initiated. Here, all the interrelationships between sensors are computed. This produces parameters which represent a given joint’s movement (example: data from sensors located on the thigh and calf are used to determine the parameters of the knee). Such a relational data model is then used to estimate data relevant to the testing personnel. The second step of data processing computes speed, acceleration, movement in space, and enables change frequency analysis. The latter one provides information, e.g., about changes in the degree of knee flexion (ψ), abduction (θ), and rotation (φ) in a given subject. The processing frequency at the first stage is guaranteed by the use of the microchip and is maintained at 200 Hz. The processing frequency at the second stage is dependant on the tested parameter and maintains the level of at least 60 Hz. Orthyo is a flexible system, as it can be adapted to achieve new functionalities and additional analyses. The archiving and retrieving system provides access to cloud-stored data and thus enables web-based tracking of results. The coordinate reference systems of the upper sensor (S1, strapped to a thigh), lower sensor (S2, calf-mounted), and the referential system (ENU) are shown in Figure 1.

### 2.3. Experimental Procedures and Instruments

The subjects were informed about the methodology of individual tests before their commencement, and underwent one control test to ensure correct understanding of instruction. Two test conductors were always present. Four sensors were used simultaneously (two per limb) along with two smartphones with Android 5.0 lollipop operating system and the installed application. Prior to measurement, test conductors logged into the application and paired the smartphones with the sensors through Wi-Fi. After establishing a connection, the sensors were calibrated. At this point, a patient profile was created, which included information such as age, weight, height, sex, and an indication of an extremity (left or right). The sensors were attached to patients’ limbs using velcro. One sensor was placed on the thigh and the other on the lower leg. Sensors were aligned along a line going through the centre of the kneecap. All measurements were performed three times with the same testing protocol.

### 2.4. Testing Procedures

**Figure 2:**
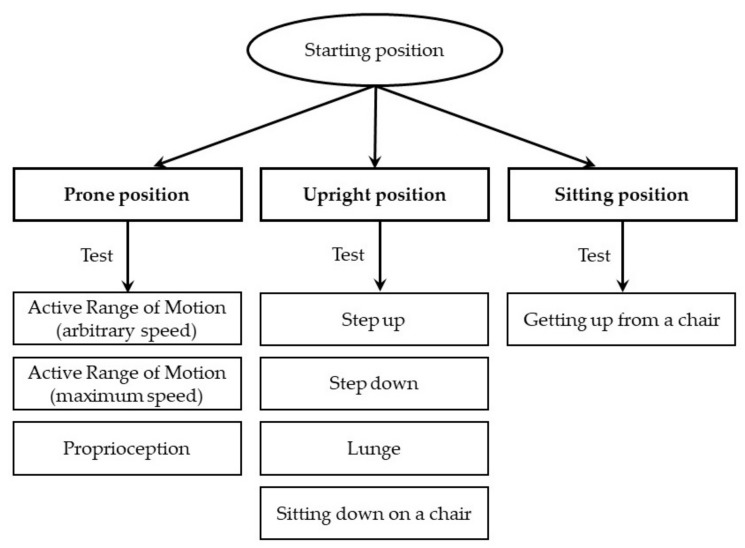
Presentation of eight tested activities.

The knee joint test consisted of eight activities (see Figure 2). During each event, the following parameters were recorded: A range of motion (degrees), angular velocity (degrees/second), and deviation from correct movement trajectory in the sagittal plane of the joint expressed by the sum of squared errors (SSE, degrees^2/seconds).

**Figure 3:**
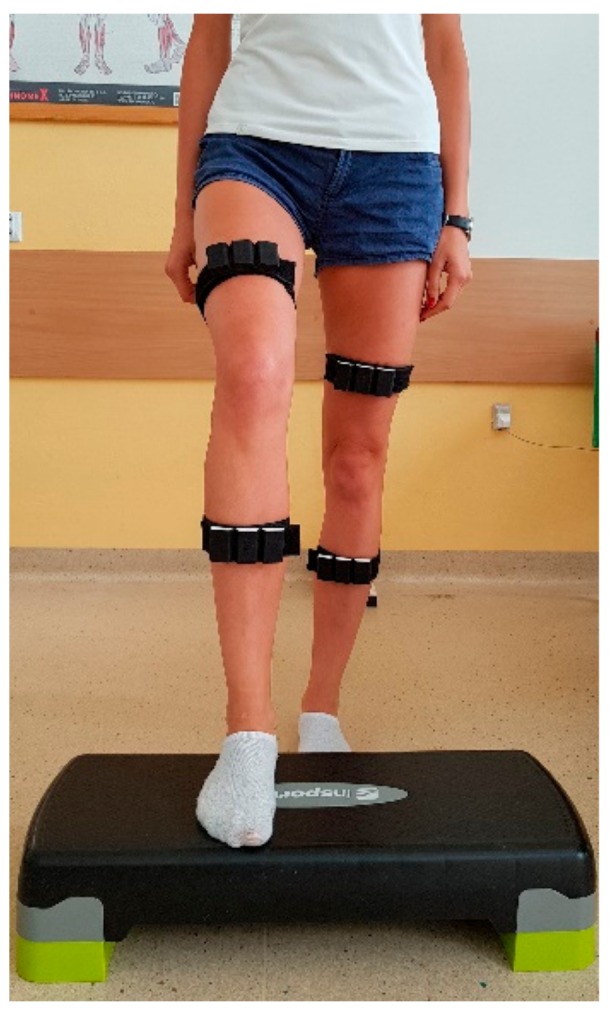
Recording in step up position.

The first test measured the active range of motion of the knee joint flexion. The subject lay on their back with hands at their sides and head lying freely on the couch. They were instructed to bend the knee joint three times with a full possible range of motion and at any preferred speed. The testing personnel stabilised the subject by placing hands on the wings of the ilium, and then “start” command was given for the subject to initiate the movement. The test was performed for both lower extremities. In the next step, that subject was asked to repeat the same test but at maximum speed. Other test elements remained the same. The next test assessed a person’s ability to return to the knee flexion set by the testing personnel and without controlling the position with eyes. For study, we arbitrarily assumed 60° flexion as the target position. The subject lay on their back with hands at sides and head lying freely on the couch. They were asked to return to the knee’s flexion degree set earlier by the therapist. Prior to the test, the personnel positioned the subject’s limb at 60° (the right angle was identified with data from the mobile application). The subject was supposed to remain at this position for 5 s and then straighten the leg. After the “start” command, the subject returned to the set point. After the mobile application measured the flexion degree, the subject straightened out the lower limb. The test was performed for both lower extremities. In the next test, subjects were standing in a relaxed, comfortable position in front of a step and with their feet aligned parallel. The distance between the feet and the step was each time established individually, as it was equal to a given subject’s foot length. Next, the subject was asked to stand on a 15-cm-high step (Figure 3). During the test, selected kinematic parameters for the lead leg were measured. The test was performed three times for both limbs. Similarly, when subjects were getting off the step, they were supposed to put their lead foot in a designated place. The distance equalled half of a subject’s foot length. The sensors recorded parameters for the trail leg. During the sixth test, subjects were asked to make a forward lunge, a movement known, e.g., from the fencing sports discipline. To carry out the test correctly, subjects were supposed to achieve a 90° flexion in the lead leg’s knee joint. The subject placed hands on her hips and looked straight in front of her. Next, the sensors recorded data for the lead leg. The last two tests recorded data for knee movement performed during sitting down and standing up from a 45 cm high chair with no back support or armrests. The subject would sit down and then stand up with her feet parallel and with 20 cm distance between them, arms were crossed over the chest and eyes looking straight. Data was captured for both lower limbs simultaneously.

### 2.5. Statistical Analysis

Data were analysed with Statistica software version 13.1. Descriptive statistics were reported as means and standard deviations (SD). The Shapiro–Wilk test was used to assess the normality of distributions in the test scores. Nonparametric analyses were used when the data did not meet the assumptions for parametric analysis. The dependent *t*-test or nonparametric Wilcoxon’s signed ranks test were conducted to compare the differences between the means of three measurements for the left and right limbs. ANOVA with repeated measures, multivariate analysis of variance, or nonparametric Friedman test were used to determine whether there were any differences between the three measurements. A post-hoc analysis was used in the cases when there were statistically significant differences in the measures. *p*-values of less than 0.05 were considered statistically significant.

## 3. Results

Comparing the mean values of the active range of motion and SSE of the knee joint performed at arbitrary and maximum speed did not reveal any significant differences between the three consecutive tests (Table 1). Furthermore, we did not find differences when comparing the mean range of motion values and SSE at both speeds between the left and right limb. Analysis of mean values of knee joints’ active motion at arbitrary and a maximum speed showed significant differences only between the values of the lower left limb’s maximum speed over the three consecutive tests. However, we did not observe differences in the mean values of arbitrary speed and maximum speed of knee joint movements between the left and right lower limb.

Analysis of flexion speed and SSE when subjects were supposed to return to the 60° knee position did not show any significant differences, neither between the three tests of the same limb nor when comparing the left with the right knee joint.

Assessment of range of motion and potential deviations from the knee’s correct trajectory at arbitrary speed when stepping up showed no significant differences between the values in the three consecutive tests, and no differences between the mean values of the left and right lower limb. Comparing the mean values of speed at which the subject entered the step, we found statistically significant differences between the three consecutive measurements of the right lower limb. A similar analysis of three consecutive test results for getting off the step revealed no statistically significant differences between knee joint’s motion ranges, motion speed, and deviations from the correct trajectory. The only relevant difference found was in the mean speed values when getting off the step between the left and the right lower limb. When testing the usability of the sensor system to capture the characteristics of the knee joint motion during a classic forward lunge, we did not find any significant differences in the mean values of active range of motion, nor in the speed at which the lunge occurred or trajectory deviations. The only difference revealed was when we compared the mean range of motion values from the three consecutive tests of the left and the right lower limbs. Results from the three sit down tests showed that the only significant difference was between flexion speeds of the right knee joint. There were no relevant differences between the results of the three stand up tests. Finally, in both of the above tests, no significant differences were found in the mean values of the range of motion, speed, and SSE between the left and the right lower limb.

## 4. Discussion

In recent years, the Internet has been used to transmit biomedical signals to make patients’ lives easier, and implementations of treatment plans more effectual [18]. First known studies that were investigating possible applications for wireless sensors attached directly to patients’ bodies to measure changes in motion kinetics were published more than ten years ago [19,20]. Measurement precision is an essential factor that requires deep consideration when investigating the diagnostic potential of wireless sensors. The accuracy of such testing methods has been confirmed by van Acht et al. [21], Chung et al. [22], Djurić-Jovičić et al. [23], and finally Chardonnens et al. [24]. The reliability of online data collection has been proved to match offline-based methods by Ermes et al. [25] The utility of mobile application used in wireless motion capture systems for physiotherapeutic purposes has been confirmed in a few studies. The analyses, however, rather assessed the range of motion of selected [26,27]. Several other studies have recommended the type of system under examination as reliable testing tools for more complex activities such as gait [23,28,29,30,31].

Until 2011, all research focused on systems in which sensors worked with a desktop computer, which we believe significantly limited the usefulness of such methods in monitoring treatment progress. Wagenaar et al. [32] first reported and underscored the potential of combining motion sensors with mobile applications to monitor the efficacy of rehabilitative and physiotherapeutic procedures. Kos et al. stated in their work that over 50% of people in the world had a smartphone in 2013. In this way, smartphones became an easily accessible device for collecting information flowing from the environment [33]. Liu and Liu [34] indicated that due to ease of use and lower costs, a system for transmitting data between sensors and a smartphone via Bluetooth might soon be of significance in monitoring rehabilitation effects. Similar conclusions have been reached by Wu et al. [35], Kouris and Koutsouris [36], and Horak et al. [37].

As in the case of our work, Joukov et al. [38] demonstrated successful assessments of selected lower limb gait movements. Gait assessment technology utilising dedicated mobile applications and wireless sensors was the study subject for other researchers as well [39,40,41]. Research into the usefulness of wireless sensors in experimental, and clinical conditions must take into account this technology’s technical considerations—a need that was met by more than a dozen compelling studies [42,43,44,45]. Courtemanche et al. [46] were the first to suggest three-dimensional (3D) printing as a means of producing sensors, and this method was used to obtain the gear for our project. Allseits et al. claimed that for measuring the range of flexion and extension of the knee, a gyroscope is a sufficient tool [47]. However, Joukov et al. [48] indicated that for a complete motion assessment and effective monitoring, it is necessary to combine an accelerometer, a gyroscope, and a motion capture tracer in a single monitoring unit. As in our method, they also used sensors that were technologically complex and advanced (see the methods chapter). We believe that the estimation of three-plane orientation without the use of accelerometer data additionally introduces an error related to orientation drift.

Analysing the literature, one can see a tendency that, for the knee joint, mainly measurements of the angle of flexion and extension are collected, which, according to the authors of these articles, are keyed in the monitoring knee function [47,49]. However, limiting yourself to only this one scope excludes the assessment of dynamic valgus described by Hewett et al. [50], which was also observed during pedalling motion by Cordillet et al. [51]. Moreover, the sensors used in our project also analysed the SSE parameter, interpreted as deviations in the frontal plane from the correct trajectory described by us in the methods section.

We believe that repeatability of measurement results is another salient factor determining the validity of wireless sensors. This was also pointed out by Chen et al. [52]. Our research project concentrated mainly on the repeatability of results in assessments of selected motor activities that engage the knee joint. The reason was that in the future, we plan to validate the measuring method under investigation as a possible tool for monitoring patients’ exercises performed in domestic settings without the presence of a physiotherapist. In the majority of cases, the measurement results we obtained in three consecutive tests of each subject were marked by high repeatability, which is evidenced by the lack of significant differences between the results for the left and right knee joint (see Table 1 and Table 2). Importantly, the repeatability of results was observed in both arbitrary and maximum speed measurements. There are no similar studies found in the relevant literature, which suggests the innovative character of our testing method. Wireless sensors monitoring patients’ activities may be utilised to verify the correctness of exercises aimed at improving the knee joint’s range of motion, or exercises which increase motion correctness and coordination at arbitrary speed applied by the patient. A diagnostic novelty offered by the investigated method is repeatable assessments of the degree of deviation from correct flexion and extension trajectory of the knee joint. To date, there have been no publications of such type. This study’s salience lies in the fact that maintaining a proper trajectory is a pre-condition for the correct function of the knee joint in many life and sports activities [53]. Furthermore, all deviations from the correct knee joint trajectory signal possible joint instability [53,54] or dissynergy of muscle activities that affect the joint [55], which could be caused by fatigue [56]. Because precise motor control and being able to feel and indicate a joint’s position are crucial elements determining proper knee function, we decided to assess patients’ ability to return to flexion of 60° degrees, the speed used when re-establishing this angle, and we tested patients for any deviations from proper flexion path. For all three examined areas, there were no significant differences found between the three performed tests, nor between results recorded for the left and right knee joint (see Table 1). Subject literature shows no wireless sensors study of such type. We are confident that in the future, this type of analysis presented will gain currency in monitoring treatment processes due to the repeatability of results and the overall mobile character of the method.

All study results presented thus far regarded isolated tests, i.e., subjects remained supine, and while moving the knee joint, any associated movements were eliminated and thus did not affect the tested knee joint functions. Besides such “isolated exercises”, a rehabilitative practice commonly employs those that engage several joints and muscle group. Therefore, we have tested a few global motor tasks, vital for people’s day-to-day activities, such as sitting down on a chair, standing up, forward lunge, and getting on and off a step. In this area, we also proved in almost all cases that the results were repeatable in three consecutive tests, which further makes us confident that the studied method is very much worth recommending for monitoring independently carried out exercises. Similar studies analysing the same global activities we have are scarce; however, authors from associated subject areas have demonstrated consistency between results produced by this study’s method and methodologies traditionally applied to assess motor abilities. Papi et al. [57] tested fourteen healthy volunteers who were instructed to do rehabilitative knee joint exercises, including a 5-time sit down/stand up test and walking on a treadmill with slow, preferred, and high speed. Results showed a high correlation (*r*^2^ > 0.7) and consistency (mean range difference: −0.02−0.03 m, 0.005–0.68 s) with those obtained in standard assessment methods. Arif and Kattan [58], on the other hand, showed that wireless sensors are useful for identifying motion orientation in real settings. To this end, they tested twelve physical activities. Classification results proved the method to be highly reliable and demonstrated significant precision and sensitivity in more than 95% of all examined physical activities. Jaysrichai et al. [59] found that the test results for knee flexion, hip and knee flexion, lunge, and gait were all highly consistent (intraclass correlation coefficient (ICC) from 0.84 to 0.99) with results obtained in tests that used the Qualisys motion capture system, which strongly suggests that the new knee joint testing method is a prospective one.

## 5. Conclusions

The presented study in healthy participants suggests that the examined testing method may be of high importance for functional assessments of the knee joint. Therefore, for our follow-up research, we are planning to use the method to diagnose people with different types of knee joint disorders. Such a study may reveal possible shortcomings of using sensors, as the patients will show some functional impairments. We also wish to examine the prospect of remotely monitoring exercise correctness. This would enable overseeing the course of rehabilitation in stages where the patient has so far remained without physiotherapist’s supervision, i.e., mostly when doing exercises alone at home.

### Limitations

At this point, a question needs to be raised about the popularity of the Android version used in the study among Polish people, as well as the potential exclusion of older generations who are more likely to use an older generation of mobile phones. Using the latest Android 5.0 lollipop version also poses the problem of general affordability. Another concern is the digital skills of people who are going to use this application at home. This study was conducted under the supervision of trained personnel who were able to assist with any issues. However, not everyone demonstrates the same set of digital skills (even simple ones such as logging into the application and entering data such as age, weight, etc. may pose a challenge). This may often be a problem regardless of age. What is more, the assistants were presenting the correct exercises to the participants, and next, they were monitoring their movements. Although the experimental setting allowed us to ensure the exercises were performed correctly, the presence of specialists might have severely affected the results.

## Figures and Tables

**Table 1:** Results for range of motion, arbitrary and maximum movement speed, and the sum of squared errors (SSE) when actively flexing the knee, and results presenting the ability to return to a given flexion (mean value ± SD).

		Left				Right				Left	Right	LvsR
Test	P.	M 1	M 2	M 3	*p*	M 1	Mt 2	M 3	*p*	Mean ± SD	Mean ± SD	*p*
Active ROM	A	125 ± 8	124 ± 8	125 ± 8	0.65	123 ± 8	122 ± 8	122 ± 9	0.06	125 ± 8	122 ± 8	0.20
	AS	179 ± 52	180 ± 46	186 ± 50	0.56	178 ± 63	169 ± 58	165 ± 52	0.10	182 ± 45	170 ± 55	0.15
	SSE	11 ± 11	11 ± 11	11 ± 11	0.65	8 ± 8	8 ± 8	8 ± 9	0.53	11 ± 11	8 ± 8	0.18
Active ROM	A	136 ± 9	135 ± 9	136 ± 11	0.65	133 ± 9	135 ± 10	135 ± 9	0.24	136 ± 9	134 ± 9	0.64
	MS	474 ± 110 ^a^	509 ± 138 ^a^	516 ± 237	0.02 *	514 ± 130	538 ± 126	511 ± 108	0.65	500 ± 142	521 ± 104	0.36
	SSE	10 ± 9	10 ± 9	10 ± 9	0.67	7 ± 7	8 ± 7	8 ± 8	0.79	10 ± 9	8 ± 7	0.35
Proprioception	A	60 ± 11	61 ± 11	60 ± 12	0.79	63 ± 10	65 ± 14	64 ± 13	0.65	61 ± 11	64 ± 12	0.16
	AS	98 ± 45	92 ± 31	93 ± 41	0.97	101 ± 44	100 ± 39	106 ± 42	0.08	94 ± 36	102 ± 38	0.20
	SSE	11 ± 9	11 ± 9	11 ± 9	0.97	12 ± 10	12 ± 10	12 ± 10	0.12	11 ± 9	12 ± 10	0.84

* *p* < 0.05, ^a^ post hoc analysis, the same letters mean significant differences; P., parameter; M 1, measurement 1; M 2, measurement 2; M 3 measurement 3; A, angle; AS, arbitrary speed; MS, maximum speed.

**Table 2:** Measurement results of selected, complex movement exercises: The range of motion, arbitrary movement speed, and potential deviations from correct movement trajectory (mean value ± SD).

		Left				Right				Left	Right	L vs. R
Test.	P.	M 1	M2	M 3	*p*	M 1	M 2	M 3	*p*	Mean ± SD	Mean ± SD	*p*
Step up	A	87 ± 12	87 ± 12	86 ± 10	0.88	70 ± 9	69 ± 8	69 ± 8	0.65	87 ± 11	69 ± 8	0.09
	S	312 ± 66	319 ± 48	313 ± 58	0.79	328 ± 56 ^a^	356 ± 59 ^ab^	313 ± 75 ^b^	0.00 *	315 ± 50	332 ± 49	0.13
	SSE	2 ± 3	2 ± 3	2 ± 3	0.08	2 ± 2	2 ± 2	2 ± 2	0.20	2 ± 3	2 ± 2	0.06
Step down	A	87 ± 12	87 ± 12	86 ± 10	0.16	83 ± 10	84 ± 10	83 ± 11	0.99	87 ± 11	83 ± 10	0.19
	S	312 ± 66	319 ± 48	313 ± 58	0.50	335 ± 67	354 ± 122	332 ± 59	0.67	315 ± 50	340 ± 55	0.02 *
	SSE	2 ± 3	2 ± 3	2 ± 3	0.91	2 ± 4	2 ± 3	2 ± 3	0.65	2 ± 3	2 ± 3	0.66
Lunge	A	99 ± 11	100 ± 13	100 ± 12	0.10	91 ± 11	92 ± 11	93 ± 11	0.63	100 ± 12	92 ± 11	0.01 *
	S	383 ± 120	364 ± 93	356 ± 82	0.79	340 ± 73	355 ± 80	365 ± 9	0.09	368 ± 88	353 ± 69	0.34
	SSE	4 ± 4	4 ± 3	4 ± 6	0.65	3 ± 6	4 ± 6	4 ± 5	0.08	4.1 ± 4	4 ± 5	0.43
Sit down	A	87 ± 9	88 ± 9	88 ± 11	0.26	85 ± 10	86 ± 10	86 ± 11	0.51	88 ± 9	86 ± 10	0.24
	S	140 ± 47	146 ± 37	146 ± 39	0.69	147 ± 42 ^c^	142 ± 40	136 ± 37 ^c^	0.02 *	144 ± 36	142 ± 37	0.64
	SSE	4 ± 6	4 ± 6	4 ± 7	0.79	4 ± 4	4 ± 6	4 ± 6	0.67	4 ± 6	4 ± 5	0.50
Stand up	A	89 ± 13.7	88 ± 14	88 ± 13	0.25	85 ± 11	86 ± 12	84 ± 13	0.35	89 ±13	85 ± 12	0.11
	S	140 ± 31	148 ± 45	137 ± 57	0.50	140 ± 31	147 ± 42	139 ± 43	0.18	142 ± 40	142 ± 35	0.99
	SSE	52 ± 34	51 ± 33	47 ± 35	0.44	56 ± 37	56 ± 37	53 ± 38	0.19	50 ± 33	55 ± 37	0.52

* *p* < 0.05, ^abc^ post hoc analysis, the same letters mean significant differences. P., parameter; M 1, measurement 1; M 2, measurement 2; M 3, measurement 3; A, angle; S, speed.
